# Proceedings of the Mississippi State Dental Association

**Published:** 1877-10

**Authors:** M. C. Marshall


### PROCEEDINGS OF THE MISSISSIPPI STATE DENTAL ASSOCIATION.

The third annual meeting of the Mississippi State Dental Association was held in Canton, Mississippi, August 15th and 16th, 1877, at the office of Dr. T. O. Payne.

The meeting was called to order by Vice President Dr. T. O. Payne, in the chair, at 11.50 o'clock A. M.

The roll having been called by Dr. A. H. Hilzhaim, acting Secretary, the following named members answered to their names, T. O. Payne, W. T. Martin, R. J. Miller, and A. H. Hilzheim, who paid their annual dues. Drs. B. Jones, J. D. Miles, A. Risir and J. B. Askew were represented by proxy.

The committee on arrangements reported, which was received.

Drs. W. H. Marshall of Oxford, Miss., and M. C. Marshall of Winona, Miss., applied for membership and were duly elected.

A letter was received from the Pres't of the Association—Dr. J. Bossken, of Vicksburg, which the Secretary was requested to spread upon the minutes.

The report was made by the Treasurer, who wrote regarding the impossibility of attending the meeting.

No report was made by the Committee on Publication.

A special report of the Committee appointed to secure the passage of an act of the Legislature regulating the practice of Dentistry in the state of Mississippi, was read and received, and the same committee consisting of Drs. A. H. Hilzheim, R. J. Miller and J. D. Miles, continued, and instructed to use every effort to secure its passage.

Accounts of Drs. Miles and Miller of expenses at Jackson,

Miss., during the last session of the Legislature allowed. Adjourned until 3 o'clock P. M.

AFTERNOON SESSION.

At 3 oclock P. M., the meeting was called to order by Dr. T. O. Payne, in the chair.

An essay by Dr. W. L. Stovall, of Winona, Miss., was read by Dr. Martin; Subject- -"Want of Professional Honor."

Dr. Martin moved that a vote of thanks be extended to Dr. Stovall for his favor, which was adopted, and the Secretary was structed to notify him of the same, and to invite him to become a member of this Association.

An essay by Dr. J. D. Miles, of Vicksburg, Miss., was read by Dr. A. H. Hilzheim:—Subject: "Professional Elevation." A vote of thanks was passed for Dr. Miles' paper and the Secretary instructed to inform him of the same.

The subject of Dental Education was discussed, and urged by Drs. W. L. Martin and W. H. Marshall. Dr. Martin thought the public should know more of Dentistry.

Dr. M. C. Marshall introduced the following resolution:

That a committee of three be appointed by the President of this Association to prepare for publication a treatise, "Educating the Public" on dental subjects, to be acted upon at their next meeting—adopted.

On motion of Dr. W. H. Marshall the Association adjourned until 9 A. M., Thursday morning.

THURSDAY MORNING, AUGUST 16TH, 1877.

Meeting called to order by Dr. T. O. Payne, at 10 o'clock.

Dr. Martin reported that as the American Dental Association met at or about the same time as the Mississippi State Dental Association, it was impossible for Dr. A. H. Hilzheim and himself to attend as delegates—report received.

A paper by Dr. G. C. Daboll, of Buffalo, N. Y., subject: "Pulp Capping" was presented and on motion of Dr. R. J. Miller was ordered to be read by Dr. Martin.

After the reading Dr. Martin moved that a vote of thanks be passed for Dr. Daboll's paper, and that the Secretary inform him of the same, also that the paper be kept among the archives of the Association, which was carried.

Dr. Daboll recommended the preservation of the nerve by capping, in preference to destroying it, by using Lacto Phosphite of Lime, or Fletcher's Artificial Dentine, as a capping. Dr. Martin thought the Lacto Phosphite of Lime as received from the Depots was not a good article for use on account of evaporation of Lactic Acid. Dr. Hilzheim recommended Fletcher's Dentine as superior to anything he had ever used for capping. Dr. T. O. Payne exhibited a specimen of nerve canal filling with Os Artificial. The use of which he explained, stating that after the nerve had been thoroughly extirpated, the Os Artificial should be prepared carefully, mixing to the consistency of cream, allowing it to run thoroughly to every part of the canal; the specimen indicated success.

Dr. Hilzheim advocated small threads of cotton as a vehicle to convey the Os Artificial to the end of the root.

Dr. Martin recommended the use of small pieces of lead to be introduced into the nerve canal, he had succeeded well in this way.

Dr. A. H. Hilzheim offered a resolution for the appointment of a committee to memoralize the Legislature to repeal the Privilege License Law as regards the Dental Profession on the ground that it is an unjust discrimination in favor of the Medical Profession, as it excuses them from paying the tax because they do "charity practice" while Dentists are really at a greater outlay in charity practice than Physicians—the resolution was unanimously adopted.

Dr. A. H. Hilzheim offered the following resolution:

*Resolved*, That the following amendments be made in the Constitution and By-Laws of the Mississippi State Dental Association.

That Section 3, Article 4, of the Constitution be so amended as to read, "the same rule in to regard balloting as in Section 1."

That Section, 2 Article 5, of By-Laws be amended, by striking out the words, "the 2nd Vice President if present, and if not."

That Section 1 of By-Laws, be amended by inserting at its close the following: It shall also be the duty of the President to deliver the annual address at the opening of the meeting.

That Article 7, of By-Laws be amended by inserting after

"Southern Dental Association" and, to such other Dental Societies as the Association may deem proper.

The following was offered by Dr. Martin, which was adopted:

*Resolved,* That the next annual meeting of this Association be held on the 2nd Wednesday in September, 1878, in order to give delegates to this Association time to attend the National Association and return.

Dr. M. C. Marshall moved that the Secretary be instructed in sending out invitations next year, for the annual meeting, to inform Dentists that a code of ethics would be presented, at said meeting, for adoption; urging all members to be present to consider the matter—motion carried.

The election of officers being in order, Dr. Martin and Dr. W. H. Marshall were appointed tellers. Dr. A. H. Hilzheim was placed in nomination and was elected by the unanimous vote of the Association, President for the ensuing year.

Drs. W. L. Martin and R. J. Miller were placed in nomination, and on the second ballot Dr. Martin was elected Vice President of the Association for the ensuing year.

Drs. R. J. Miller, W. H. Marshall and M. C. Marshall were nominated for Secretary and Treasurer. Dr. M. C. Marshall was elected on fourth ballot.

Dr. W. H. Marshall moved an adjournment until 2½ o'clock P. M., carried.

Association assembled at 3 P. M. President Hilzheim in the chair.

The President appointed an Executive Committee consisting of the following named gentlemen: Drs. R. J. Miller of Durant, chairman, W. H. Marshall of Oxford, and E. E. Spinks, of Meridian. Also committe to prepare a treatise on Dentistry for public distribution consisting of Drs. J. D. Miles of Vicksburg, chairman, W. L. Martin of Yazoo, A. Riser of Port Gibson.

The place of the next meeting of the Association was now before the meeting. Winona, Montgomery Co., was selected, and the following gentleman appointed committee of arrangements: Drs. M. C. Marshall of Winona, chairman, R. J. Miller of Durant, and W. H. Marshall of Oxford.

Dr. W. H. Marshall donated to the Association a microscope,

Dr. T. O. Payne moved a vote of thanks to Dr. Marshall, which was carried unanimously.

On motion of Dr. Payne a committee consisting of the follow-named gentleman: Drs. W. H. Marshall of Oxford, chairman, Askew of Vicksburg, and Payne of Canton, were appointed to solicit microscopical objects from Dr. S. P. Cutler.

Dr. W. H. Marshall was appointed Assistant Secretary.

Dr. R. J. Miller, chairman of Executive Committee reported the following named gentleman who had been solicited to write essays, to be read at the next regular meeting.

Dr. A. H. Hilzheim of Jackson, Dr. E. E. Spinks of Meridian, and Dr. J. B. Askew of Vicksburg.

Dr. W. H. Marshall and M. C. Marshall were appointed delegates to the Southern Dental Association, which meets in Atlanta, Georgia, August 1878. Drs. A. H. Hilzheim and J. D. Miles were appointed delegates to the American Dental Association.

Drs. J. B. Askew and E. E. Spinks were appointed delegates to the American Dental Convention.

Dr. W. H. Marshall moved to adjourn until the next regular meeting, carried. M. C. MARSHALL,

*Secretary and Treasurer.*

