# Correction

**Published:** 1877-11

**Authors:** 


### EDITORIAL.

### CORRECTION.

In the proceedings of the Mississippi Dental Association, published in the October No. of the REGISTER, page 437, twelfth line from the bottom, the name J. Bosskin, should be J. B. Askew.

